# The case for compulsory licensing during COVID-19

**DOI:** 10.7189/jogh.10.010358

**Published:** 2020-06

**Authors:** Hilary Wong

**Affiliations:** University of California, Berkeley, School of Law, USA

Over the past few months, the COVID-19 pandemic has devastated industrialized countries in Asia, Europe, and North America. The outbreak will inevitably escalate in developing countries as well. While there is yet to be a proven cure or treatment for COVID-19, commonly referred to as the coronavirus, researchers are racing to test new and existing drugs in search of an effective panacea. As governments of developing countries ramp up efforts to fight the virus, they must take measures not only to contain the virus but also to ensure that COVID-19 treatments will be accessible and affordable following discovery.

Even in wealthy countries such as the United States, many COVID-19 patients have struggled with the cost of treatment, especially those who have suffered from severe COVID-19 and its subsequent complications [1]. COVID-19 patients are likely to face even more challenges accessing and affording medical treatment in developing countries, especially low-income countries with under-resourced healthcare systems. Furthermore, many individuals in low-income countries may be at high-risk of suffering severe COVID-19 because of weak lungs or compromised immune systems from chronic malnutrition, tuberculosis, or HIV.

In anticipation of the needs of their most vulnerable populations, governments of developing countries should prepare to issue compulsory licenses of any effective COVID-19 treatments. Compulsory licensing, a provision in the Agreement on Trade Related Aspects of Intellectual Property Rights (“TRIPS Agreement”), enables governments to supply its citizens with generic versions of patented treatments either through domestic production or foreign imports. As will be discussed below, compulsory licensing was used as a successful policy tool for improving access to antiretroviral drugs in the face of the AIDS epidemic. While there is no approved treatment for COVID-19 at present, national governments are legally entitled to issue compulsory licenses and should not shy away from this policy option when a treatment is available.

## THE TRIPS AGREEMENT AND COMPULSORY LICENSING

Signed concurrently with the establishment of the World Trade Organization (WTO) in 1994, the TRIPS Agreement sought to create a global intellectual property rights regime that would harmonize legal standards across WTO member states [2]. Prior to the TRIPS Agreement, many countries excluded pharmaceutical products from patentability in order to ensure drug prices were affordable. India and Brazil, for example, had previously only allowed for process patents and not product patents [3]. This permitted domestic generic pharmaceutical companies to reverse-engineer and produce bioequivalent drugs.

Under a TRIPS-compliant patent regime, WTO member states are required to guarantee product patents and exclusive marketing rights to innovators. These expanded patent protections enable pharmaceutical companies to set high prices, which help companies recoup R&D costs but also often put medicines out of reach for the poor. For this reason, former World Bank Chief Economist Joseph Stiglitz has described the TRIPS Agreement as “a death warrant for thousands of people in the poorest countries of the world” [4].

The TRIPS Agreement, however, provides governments with some flexibility in managing patents for public goods such as pharmaceuticals. In particular, the Doha Declaration on the TRIPS Agreement and Public Health in November 2001 highlights and clarifies a key flexibility afforded to countries in Article 31 of the TRIPS agreement: the right to grant compulsory licenses. Compulsory licensing refers to the use of a patent without the authorization of the patent holder. Specifically, issuing a compulsory license for a pharmaceutical treatment allows a government to locally manufacture or import generic versions of the treatment without the patent holder’s consent.

Clause 5 of the Doha Declaration reaffirmed that “each [WTO] [m]ember has the right to grant compulsory licenses and the freedom to determine the grounds upon which such licenses are granted” [5]. Additionally, in situations of “national emergencies” and “other circumstances of extreme urgency,” governments can issue compulsory licenses without normal requirements, such as negotiating with the patent holder [6]. Clause 5(c) further clarified that: “public health crises, including those relating to HIV/AIDS, tuberculosis, malaria and other epidemics” can constitute “a national emergency or other circumstances of extreme urgency.” There can be no doubt that the COVID-19 pandemic is a public health crisis within the meaning of clause 5(c) that justifies the use of compulsory licenses.

There are several countries that have successfully used compulsory licenses to provide their citizens with affordable medicines during past public health crises. Below is a snapshot of their experiences.

## PAST EXPERIENCES: COMPULSORY LICENSES FOR ANTIRETROVIRAL TREATMENTS

Approximately twenty countries have either issued or publically entertained issuing a compulsory license for one or more pharmaceutical products since the founding of the WTO [7]. In some cases, governments did not end up issuing a compulsory license. A mere public announcement or discussion of potentially issuing a compulsory license for a drug has sometimes led the patent holder to offer a discount or a voluntary license for the drug.

Thus far, compulsory licensing – whether ending in an actual issuance or a lower negotiated drug price – has largely been for HIV/AIDS related treatments. In the 2000s, Brazil, Ecuador, Ghana, Indonesia, Malaysia, Mozambique, Thailand, Rwanda, Zambia, and Zimbabwe each issued compulsory licenses for one or more antiretroviral drugs to respond to the plight of their HIV-infected citizens who could not afford antiretroviral therapy. While most countries issued licenses for a specific patented drug, Ghana and Zimbabwe issued categorical compulsory licenses on all antiretroviral drugs [7].

Two countries that had notable success with decreasing the price of antiretroviral drugs in the mid-2000s were Thailand and Brazil. Both countries provided free antiretroviral treatments to all citizens living with HIV/AIDS and were thus keenly motivated to seek out affordable antiretroviral supplies. Specifically, both sought to procure and provide efavirenz (marketed as Sustiva by Merck) and lopinavir/ritonavir (marketed as Kaletra by AbbVie, then Abbott Laboratories) to their patients. In initial price negotiations with Thailand, Merck offered efavirenz at the price of US$500 per patient per year (PPPY), and Abbott offered lopinavir/ritonavir at the price of US$2200 PPPY. The Thai government rejected both of these offers due to the high prices and issued compulsory licenses for both drugs in late 2006 and early 2007. These licenses allowed the Thai government to import generic versions of the antiretroviral drugs from India at a significantly lowered cost – generic efavirenz at US$224 PPPY and generic lopinavir/ritonavir at US$676 PPPY [8].

Like Thailand, Brazil issued a compulsory license for efavirenz in 2007. Merck initially offered efavirenz to Brazil at the price of US$760 PPPY. By issuing a compulsory license for efavirenz, Brazil was able to generically import efavirenz at US$170 per patient/y. However, unlike Thailand, Brazil did not issue a compulsory license for lopinavir/ritonavir. In response to Brazil’s persistent price negotiations and credible threat of using a compulsory license, Abbott Laboratories eventually lowered the price of lopinavir/ritonavir in Brazil from US$3241 PPPY to a price of US$1380 PPPY for an older version and US$1518 for a heat-stable version [8]. Notably, the discounted price of Abbott’s lopinavir/ritonavir in Brazil was still more than twice the price of the generic lopinavir/ritonavir that Thailand acquired through compulsory licensing and importing from India.

**Figure:**
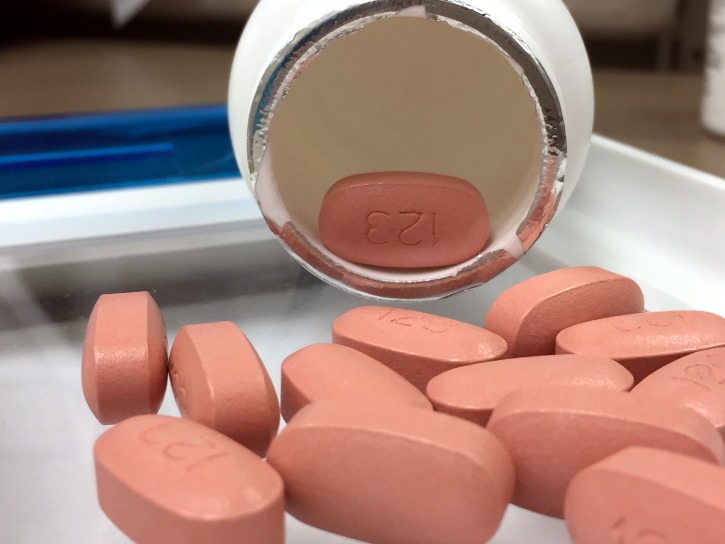
Photo: HIV antiretroviral medication by NIAID on Wikimedia Commons.

Despite the opportunity to lower drug prices, many low-income countries tend to shy away from using compulsory licenses for pharmaceutical products. Admittedly, some countries are bound by restrictions in bilateral relations such as free-trade agreements with provisions limiting the use of compulsory licenses. However, many countries are not legally restricted from compulsory licensing but avoid doing so due to fears of trade retaliation and the complicated nature of imposing compulsory licenses [9]. Such barriers are difficult but not insurmountable.

In the case of Thailand, domestic and international public support helped to dampen the retaliatory responses of Western governments as well as pharmaceutical companies that threatened to withdraw new products from the Thai market. To navigate the complicated nature of imposing compulsory licenses, Thai officials diligently educated themselves on the TRIPS Agreement and the Doha Declaration. Notably, conferences with the WHO and non-governmental organizations such as Médecins Sans Frontières and Knowledge Ecology International helped to strengthen Thai officials’ capacity to deploy compulsory licenses and TRIPS flexibilities [10].

The experiences learned from the HIV/AIDS epidemic should be applied to the fight against COVID-19. Countries that previously used compulsory licenses to provide affordable antiretroviral drugs for their citizens should do so again if necessary. Developing countries that have not previously used compulsory licensing should leverage the experiences of countries that have. The WHO, WTO, and legal organizations such as the Advisory Centre on World Trade Organization Law (ACWL) can facilitate knowledge sharing among public health officials and government lawyers across developing countries. Whether it is through virtual workshops or other collaborative online forums, now is an important time to help developing countries build the legal and logistical capacity to use compulsory licensing in the face of COVID-19.

## COMPULSORY LICENSING IN THE TIME OF COVID-19

Several countries have already publically considered compulsory licensing as part of their COVID-19 response. On March 24, 2020, Israel issued a compulsory license to import generic versions of lopinavir/ritonavir (AbbVie’s Kaletra). The Israeli Ministry of Health has determined that the antiretroviral drug could be a possible treatment for COVID-19 patients. Unlike Thailand and Brazil, Israel did not issue the license due to the drug’s pricing. Instead, Israel issued the compulsory license and turned to generic alternatives from India because AbbVie was unable to provide sufficient supplies of lopinavir/ritonavir. AbbVie has announced that it will not enforce its patent in light of the current pandemic [11].

In order for a government to use compulsory licensing for COVID-19 related purposes, its domestic laws must have procedures in place to authorize such government action. Several countries have already taken legislative steps to ensure their governments can swiftly issue compulsory licenses as part of their COVID-19 response.

In March, legislatures in Canada, Chile, and Ecuador laid the legal groundwork for the issuance of compulsory licenses to address COVID-19. Canada’s COVID-19 Emergency Response Act amended the Canadian Patent Act to allow for a speedier process for issuing a compulsory license on public health grounds. The amendment allows the government to issue a license for necessary innovations and to negotiate remuneration later [12]. Chile’s Chamber of Deputies (the lower house of its Congress) has passed a resolution granting the use of compulsory licenses for the prevention and treatment of COVID-19. Specifically, the resolution declares that the coronavirus pandemic constitutes sufficient justification to grant compulsory licenses for COVID-19-related technologies [13]. Similarly, a Committee of the National Assembly in Ecuador has passed a resolution requiring the Ecuadorian President and Minister of Health to provide free or affordable access to COVID-19-related preventative, diagnostic, and treatment technologies through the use of compulsory licenses [14]. Other developing countries should take similar legal and legislative steps to establish a framework for using compulsory licenses in case it is necessary.

Ultimately, compulsory licensing may not be necessary. A COVID-19 cure may turn out to be an existing drug that is no longer patented. Even if the discovered cure is patent-protected, there may be drug donations, discounts, or the patent holder may offer voluntary licenses at affordable rates.

However, in case compulsory licenses do become necessary when a cure is available, countries should take the appropriate legislative steps to prepare as soon as possible.

## CONCLUSION

Compulsory licensing is a powerful public health tool – it can be instrumental for alleviating insufficient supplies of necessary pharmaceuticals as well as mitigating prohibitively expensive drug prices. Conceivably, countries may face both problems when a COVID-19 cure is ushered to market. While the rewards of patent protection are necessary to support continual innovation, the compulsory licensing exception exists for public health emergencies such as the current COVID-19 crisis. Governments must do what is necessary to fight the present pandemic. International organizations can play a key role by providing the legal know-how as well as setting a supportive tone for using compulsory licensing. In the process, pharmaceutical companies and G20 countries should not deter or retaliate against developing countries pursuing such public health measures in the time of a pandemic.
